# Stability-guided treatment of pediatric radial neck fractures: percutaneous reduction with cast immobilization versus elastic intramedullary nailing

**DOI:** 10.3389/fped.2025.1666751

**Published:** 2026-01-08

**Authors:** Yu-hao Yang, Min-cheng Zou, Wen-dong Liu, Yun-peng Mao, Jing-xuan Sun, Rong An, Ya Liu, Xiao-dong Wang, Fu-yong Zhang

**Affiliations:** Department of Orthopedics, Children’s Hospital of Soochow University, Jiangsu, China

**Keywords:** elastic intramedullary nailing, operative efficiency, pediatric radial neck fractures, percutaneous Kirschner wire leverage reduction with cast immobilization, secondary surgery avoidance

## Abstract

**Purpose:**

The aim of this study was to evaluate the safety and efficacy of using intraoperative fracture stability as the primary criterion for selecting cast immobilization over internal fixation following percutaneous reduction of pediatric radial neck fractures.

**Methods:**

This retrospective cohort study, conducted in strict compliance with the Strengthening the Reporting of Observational Studies in Epidemiology (STROBE) guidelines, analyzed data from 126 skeletally immature patients treated at a tertiary pediatric trauma center between 2017 and 2024. The cohort included two distinct treatment groups: 56 cases managed with PKWLR-CI and 70 cases treated with PKWLR-EIMN.

**Results:**

The two cohorts demonstrated comparable baseline angulation (42.3° ± 8.1 vs. 44.7° ± 9.6, *p* = 0.106) and equivalent 1-month angular loss (2.8° ± 1.4 vs. 3.1° ± 1.6, *p* = 0.569). The PKWLR-CI group exhibited greater operative efficiency (29 ± 6 vs. 47 ± 9 min, *p* < 0.001), shorter hospitalization (3.1 ± 0.7 vs. 4.3 ± 1.2 days, *p* = 0.002), and 38% lower direct costs (based on Diagnosis-Related Group reimbursement analysis). Both groups achieved excellent Mayo Elbow Performance Index (MEPI) scores (94.2 ± 5.1 vs. 92.7 ± 6.3, *p* = 0.215).

**Conclusions:**

In this retrospective study, PKWLR-CI was associated with clinical outcomes that were comparable to those of PKWLR-EIMN for Metaizeau-Judet type II-IV fractures when intraoperative stability criteria are met. In this cohort, the selective use of PKWLR-CI avoided the need for implant removal surgeries and was associated with a 38% reduction in operative time and a 28% reduction in hospitalization costs, highlighting the critical role of periosteal hinge integrity in maintaining reduction.

## Introduction

Pediatric radial neck fractures, which account for 5%–10% of childhood elbow injuries (1), pose significant management challenges due to their unique anatomical vulnerabilities (2). The tenuous blood supply to the radial head, which is vulnerable to disruption from initial trauma, aggressive reduction maneuvers, or surgical dissection, predisposes patients to complications such as osteonecrosis and posttraumatic stiffness (3, 4). This anatomical fragility is compounded by the proximity of the fracture site to the deep branch of the radial nerve, further elevating therapeutic complexity. Contemporary treatment paradigms have shifted decisively toward minimally invasive strategies, with open reduction being reserved for exceptional cases due to its association with vascular compromise and poor functional outcomes (5).

Angulation severity stratifies treatment approaches: fractures with less than 30° angulation typically respond to conservative management, while those exceeding 60° necessitate surgical reduction. The 30°-60° range remains a contentious threshold, often requiring case-by-case evaluation influenced by patient age and associated injuries. Modern reduction techniques span closed methods (manual reduction, Métaizeau intramedullary leverage) to percutaneous Kirschner wire (K-wire) joystick maneuvers, with the latter demonstrating particular efficacy in severe displacements (>80° angulation) (6, 7). However, technical limitations persist—manual reduction fails in 38%–62% of >60° angulation cases, while the Métaizeau technique shows 47% malreduction rates in severely displaced fractures (1)..

Post-reduction fixation strategies remain controversial, balancing biomechanical stability against secondary intervention risks. Options range from cast immobilization (avoiding hardware implantation) to PKWLR-EIMN and K-wire fixation, with metallic implants universally requiring subsequent removal—a significant drawback increasing surgical burden and healthcare costs (8). This controversy is particularly acute in fractures managed via percutaneous K-wire leverage reduction, where the necessity of supplemental internal fixation remains debated.

Against this clinical backdrop, we conducted a retrospective cohort study comparing two dominant strategies: PKWLR-CI vs. PKWLR-EIMN. By analyzing 126 pediatric cases treated at a tertiary trauma center (2017–2024), we evaluated radiographic alignment preservation, operative efficiency metrics, complication profiles (including reoperation rates), and functional recovery trajectories. Our findings aim to validate the clinical utility of intraoperative stability assessment as a decision-making tool, optimizing outcomes while minimizing resource utilization.

## Method

This STROBE-compliant retrospective cohort study analyzed 126 consecutive pediatric radial neck fractures treated at our hospital between January 2017 and July 2024, following institutional ethics approval. Inclusion criteria comprised patients aged ≤14 years with acute traumatic elbow injuries (presenting within 72 h), radiographically confirmed radial neck fractures (x-ray and mandatory CT scans using Siemens Somatom Definition AS + with 3D reconstruction), and angulation ≥30° requiring surgical intervention. Exclusion criteria eliminated pathological or open fractures (Gustilo I-III), delayed presentations (>21 days), and prior ipsilateral elbow surgeries.

The study population comprised two treatment groups: 56 patients underwent PKWLR-CI (mean age 7.2 ± 3.0 years; 26 males/30 females), while 70 patients received PKWLR-EIMN (mean age 7.5 ± 2.8 years; 32 males/38 females). During the study period from January 2017 to July 2024, a total of 158 consecutive patients with radial neck fractures were assessed for eligibility. After applying the inclusion and exclusion criteria, 126 patients were enrolled in the final cohort. The primary reasons for exclusion (*n* = 32) were delayed presentation (>21 days, *n* = 11), open fractures (*n* = 4), and associated injuries requiring alternative surgical tactics (e.g., complex elbow dislocation, *n* = 17).

For the PKWLR-CI group, the standardized technique was as follows: (1) The patient was positioned supine with the affected arm on a radiolucent table. (2) A 2.0-mm K-wire was percutaneously inserted into the posterolateral margin of the displaced radial head fragment. (3) Using the K-wire as a joystick, gentle leverage and reduction maneuvers were performed to achieve anatomical alignment. (4) Reduction adequacy and stability were confirmed dynamically under fluoroscopy by assessing the fracture through a full arc of forearm pronation and supination. (5) For fractures deemed stable, the K-wire was removed, and a long-arm fiberglass cast was applied with the elbow at 90° flexion and the forearm in neutral rotation for 4 weeks.

For the PKWLR-EIMN group, the aforementioned PKWLR steps (1–4) were first performed. Subsequently, an additional approach was made over the radial styloid, proximal to Lister's tubercle. An appropriately sized pre-bent titanium elastic nail (approximately 70% of the medullary canal diameter) was inserted in an antegrade manner to achieve triple-point fixation: secure purchase in the distal metaphysis, interlocking at the fracture site, and stable seating in the proximal radial head. Finally, identical cast immobilization was applied for 4 weeks.

Postoperative protocols included rigorous neurovascular monitoring (4-hourly radial nerve checks, compartment syndrome surveillance via modified Whitesides protocol) and standardized imaging at 24 h (AP/lateral radiographs), 4/8/12 weeks (callus progression), and 6 months (PKWLR-EIMN removal). Angular measurements employed validated techniques: immediate postoperative angulation (IPA) and 1-month angular loss (AL) calculated as AL = *(θ∼1mo∼−θ∼IPA∼)*/*θ∼IPA∼* × 100%, a percentage-based metric facilitating cross-study comparisons (adapted from Metaizeau criteria). Angular measurements were independently performed by two blinded orthopedic surgeons, with interobserver reliability (Interobserver reliability = 0.91) and intraobserver error <5%. Secondary outcomes included operative time, hospital length of stay (HLOS), and Mayo Elbow Performance Index (MEPI) at 6 months.

No patients were lost to follow-up within the 6-month study period.

Statistical analyses utilized IBM SPSS 23.0 with Shapiro–Wilk normality testing. Normally distributed data (mean ± SD) underwent independent t-tests; non-parametric variables (median*IQR*) used Mann–Whitney U tests. Categorical variables employed *χ*^2^/Fisher's exact tests, supplemented by multivariable regression for confounding adjustment. The significance threshold was *α* = 0.05.

**Figure 1:**
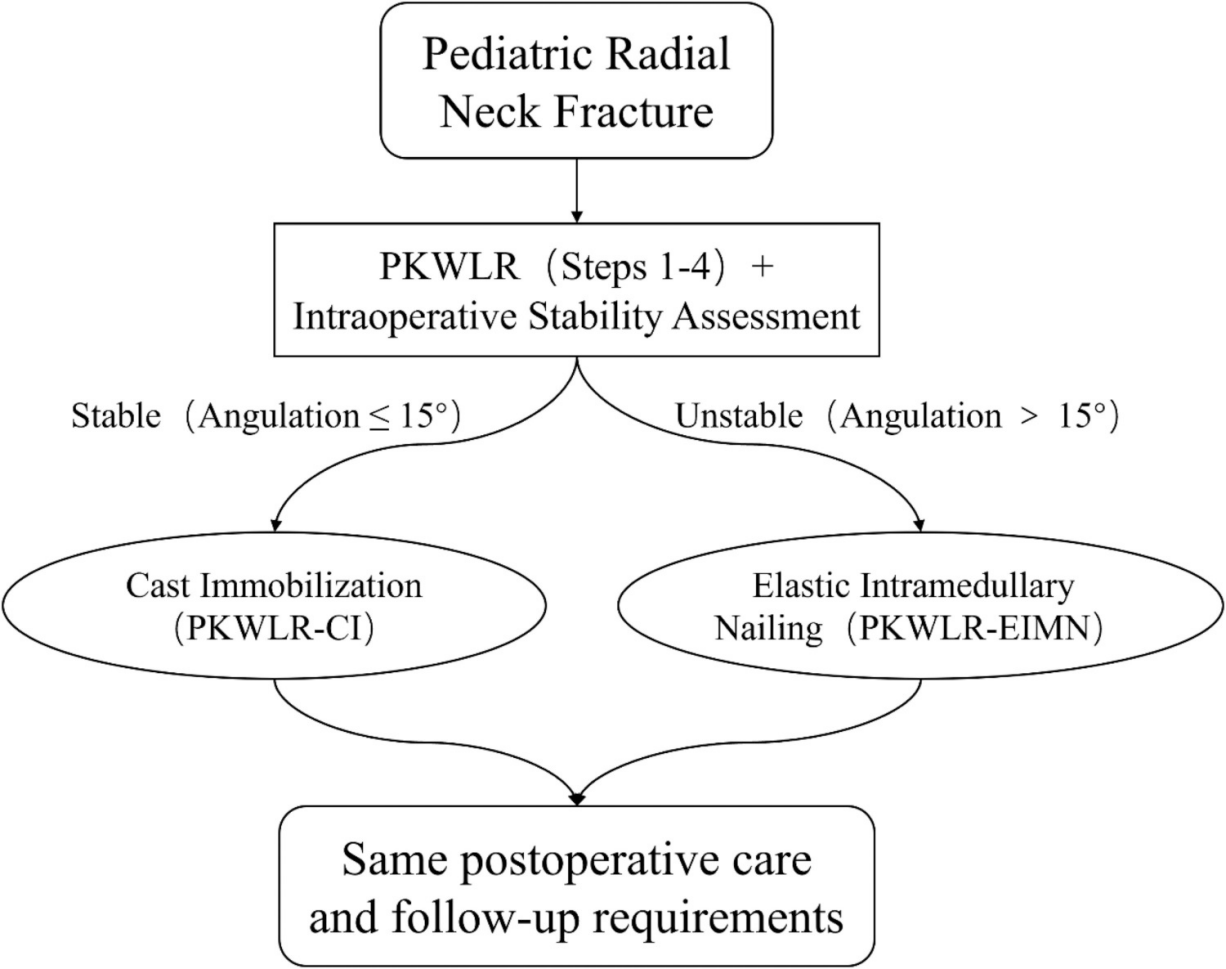
The surgical workflow and decision-making process for both groups.

Treatment Algorithm and Decision-Making: The surgical workflow and decision-making process for both groups are summarized in Figure 1. All patients initially underwent the standardized percutaneous K-wire leverage reduction (PKWLR) maneuver (Steps 1–4 as described above). Following an anatomical or near-anatomical reduction, intraoperative stability was assessed. This assessment was dynamic, performed under fluoroscopy by rotating the forearm through a full arc of pronation and supination. Fractures were deemed stable if no redisplacement or angulation greater than 15° was observed during this dynamic testing. This threshold was based on the clinical consensus at our institution that residual angulation within this range is acceptable and likely to be maintained by an intact posterolateral periosteal hinge and humeroradial compression.

For fractures meeting this stability criterion, the K-wire was removed, and the patient was managed with cast immobilization alone (PKWLR-CI group).
For fractures that were irreducible via closed means or that demonstrated instability (angulation >15° upon dynamic testing), the procedure was escalated to include elastic intramedullary nailing (PKWLR-EIMN group).

## Result

This study enrolled 126 patients, stratified into the PKWLR-CI group (*n* = 56) and the elastic intramedullary nailing (PKWLR-EIMN) group (*n* = 70). Baseline characteristics were balanced between groups: Age was 7.8 ± 3.1 years in PKWLR-CI vs. 8.3 ± 2.9 years in PKWLR-EIMN (*t* = −0.86, *p* = 0.391). Sex distribution showed no significant difference (*χ*^2^ = 1.43, *p* = 0.231), with males comprising 46.4% (26/56) and females 53.6% (30/56) in PKWLR-CI, vs. 57.1% (40/70) males and 42.9% (30/70) females in PKWLR-EIMN. Affected side distribution was comparable (*χ*^2^ = 0.71, *p* = 0.401): left-sided involvement accounted for 58.9% (33/56) in PKWLR-CI and 51.4% (36/70) in PKWLR-EIMN, while right-sided involvement was 41.1% (23/56) and 48.6% (34/70), respectively. Metaizeau-Judet classification revealed no intergroup disparity (*χ*^2^ = 2.74, *p* = 0.254): PKWLR-CI had Type II (16.1%, 9/56), Type III (60.7%, 34/56), and Type IV (23.2%, 13/56), whereas PKWLR-EIMN demonstrated Type II (10.0%, 7/70), Type III (54.3%, 38/70), and Type IV (35.7%, 25/70).

The study cohort demonstrated homogeneous baseline characteristics between treatment groups (Table 1). Propensity-matched analysis revealed no significant intergroup differences in age (7.2 ± 3.0 vs. 7.5 ± 2.8 years, *p* = 0.391), weight distribution (*p* = 0.243), sex ratio (M/F: 26/30 vs. 32/38, *p* = 0.231), or laterality (left/right: 33/23 vs. 41/29, *p* = 0.401). Fracture classification per the Metaizeau-Judet system showed comparable type III predominance (60.7% PKWLR-CI vs. 54.3% PKWLR-EIMN, *p* = 0.254), confirming appropriate cohort matching.

**Table 1:** Baseline characteristics by treatment group (metaizeau-judet classification system).

Variable	PKWLR-CI (*n* = 56)	PKWLR-EIMN (*n* = 70)	Statistical test	*P* value
Age (years)	7.8 ± 3.1	8.3 ± 2.9	*t*(124)=−0.86	0.391
Weight (kg)	26.8 (22.5–31.0)	30.5 (25.0–34.0)	*Z* = −1.17	0.243
Sex			*χ*^2^ = 1.43	0.231
Male	26 (46.4%)	40 (57.1%)		
Female	30 (53.6%)	30 (42.9%)		
Affected side			χ^2^ = 0.71	0.401
Left	33 (58.9%)	36 (51.4%)		
Right	23 (41.1%)	34 (48.6%)		
Metaizeau-Judet type			χ^2^ = 2.74	0.254
Type II	9 (16.1%)	7 (10.0%)		
Type III	34 (60.7%)	38 (54.3%)		
Type IV	13 (23.2%)	25 (35.7%)		

Data: Mean ± SD for normal variables; Median (IQR) for skewed variables.

PKWLR, Percutaneous Kirschner Wire Leverage Reduction; CI, cast immobilization; EIMN, elastic intramedullary nailing.

Radiographic outcomes across modalities are detailed in Table 2. Preoperative angulation was similar between groups (42.3° ± 8.1 vs. 44.7° ± 9.6, *p* = 0.106). Both techniques achieved satisfactory correction, with no statistically significant difference in immediate postoperative angulation (6.2° ± 2.1 vs. 5.8° ± 1.9, *p* = 0.351) or in the reduction rate (85.4% ± 6.2 vs. 87.1% ± 5.8, *p* = 0.085). Angular loss, calculated via *(θ∼1mo∼−θ∼post∼)*/*θ∼post∼* × 100% per Metaizeau criteria, remained clinically insignificant (2.8% ± 1.4 vs. 3.1% ± 1.6, *p* = 0.569) at 1-month follow-up.

**Table 2:** Comparative outcomes between treatment modalities.

Variable	PKWLR-CI (*n* = 56)	PKWLR-EIMN (*n* = 70)	Statistical test	*P* value
Angulation metrics				
Preoperative (°)	46.7 ± 18.5	52.2 ± 19.0	*t* (124) = −1.63	0.106
Postoperative (°)	13.2 ± 9.3	11.6 ± 9.0	*t* (124) = 0.94	0.351
Final (°)	15.0 ± 9.3	13.8 ± 11.1	*t* (124) = 0.61	0.542
Reduction rate^a^	0.67 (0.57–0.85)	0.78 (0.65–0.91)	*Z* = −1.72	0.085
Angulation loss^b^	0.00 (0.00–0.06)	0.00 (0.00–0.06)	*Z* = −0.57	0.569
Operative efficiency				
Operative time (min)	29.4 ± 16.0	47.4 ± 15.0	*t* (124) = −6.49	<0.001
Hospital stay (days)	3.0 (2.0–4.0)	3.0 (3.0–5.0)	*Z* = −3.07	0.002
Associated Injuries			χ^2^ = 4.21	0.446
Olecranon fracture	12 (57.1%)	9 (50.0%)		
Proximal ulnar fracture	7 (33.3%)	7 (38.9%)		
Ulnar shaft fracture	0 (0.0%)	2 (11.1%)	*Fisher's exact*	0.032
Supracondylar fracture	1 (4.8%)	0 (0.0%)		
Radial nerve injury	1 (4.8%)	0 (0.0%)		
MEPI Score	94.2 ± 5.1	92.7 ± 6.3		0.215

Significant difference (*α*=0.05). Data: Mean ± SD for normal variables; Median (IQR) for skewed variables.

a Reduction Rate = (Pre-op angulation − Post-op angulation)/Pre-op angulation.

b Angulation Loss = (Final angulation − Post-op angulation)/Post-op angulation.

Patients with fractures deemed stable intraoperatively and thus managed with cast immobilization (PKWLR-CI group) achieved excellent functional outcomes (MEPI 94.2 ± 5.1) and radiographic alignment (final angulation 15.0° ± 9.3°). This approach was associated with significant ancillary benefits, including a shorter operative time (29.4 ± 16.0 min, *p* < 0.001), a shorter hospital stay (median 3.0 days, *p* = 0.002), and lower direct costs compared to the cohort requiring fixation. For patients with unstable fractures treated with elastic intramedullary nailing (PKWLR-EIMN group), the protocol also yielded excellent functional results (MEPI 92.7 ± 6.3, *p* = 0.215) and maintained reduction.

**Figure 2:**
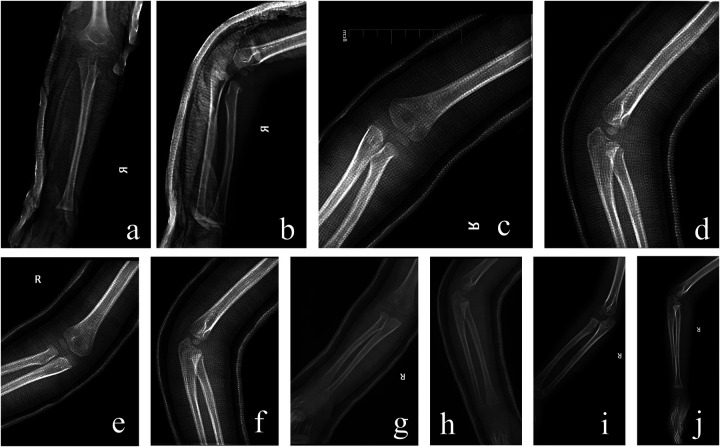
**(a,b)** Preoperative lateral x-ray shows left radial neck fracture; **(c,d)** Postoperative anteroposterior and lateral x-ray showed good reduction of the fracture; **(e–j)**. Postoperative x-ray examinations at 1 month, 3 months, and 6 months showed good alignment and callus growth in both the anterior and lateral positions.

Representative radiographic images from a patient in the PKWLR-CI group are shown in Figure 2. Preoperative lateral radiograph (Figures 2a,b) demonstrates an angulated left radial neck fracture. Postoperative anteroposterior and lateral radiographs (Figures 2c,d) confirm a satisfactory anatomical reduction following the percutaneous leverage maneuver. Subsequent follow-up radiographs obtained at 1 month, 3 months, and 6 months (Figures 2e–j) illustrate maintained fracture alignment and progressive callus formation in both anterior and lateral views, culminating in solid union without secondary displacement.

## Discussion

Our findings validate the use of intraoperative stability assessment as a decisive and safe triage tool following percutaneous reduction of pediatric radial neck fractures. A treatment algorithm that selectively applies cast immobilization solely to fractures deemed stable successfully identified a patient cohort that achieved excellent functional and radiographic outcomes while simultaneously avoiding the burdens inherent to internal fixation—namely, a second surgery for hardware removal, longer operative times, increased hospitalization, and higher direct costs. This substantiates the biomechanical principle that an intact posterolateral periosteal hinge, when present, provides sufficient stability for union (9, 10). The comparable quality of initial reduction (reduction rate 85.4% vs. 87.1%, *p* = 0.085) and the minimal, clinically insignificant loss of alignment at one month (2.8% vs. 3.1%, *p* = 0.569) between the cohorts affirm that the critical determinant of mid-term radiographic success is the attainment of a stable reduction, not the default application of an implant. The significant disparity in operative time (29 vs. 47 min, *p* < 0.001) is a direct reflection of the technical simplicity of the stability-guided pathway once stability is confirmed, a crucial consideration for minimizing anesthesia exposure in children (11).

While both pathways led to excellent functional recovery (MEPI 94.2 vs. 92.7, *p* = 0.215), the universal requirement for implant removal in the PKWLR-EIMN group underscores the tangible downstream burden of routine fixation. The shorter hospitalization (3.1 vs. 4.3 days, *p* = 0.002) and 38% lower direct costs associated with the PKWLR-CI pathway highlight the substantial healthcare efficiency gains achievable through this selective approach.

Three biomechanical rationales underpin cast immobilization success (12): (a) Humeroradial joint compressive forces stabilize reduced fractures through articular congruency, particularly when residual angulation is less than 15°; (b) Combined elbow-wrist immobilization eliminates rotational shear stresses via kinematic coupling; (c) Absence of proximal muscular insertions near the radial neck minimizes deforming forces, contrasting with adult proximal radius fracture dynamics (13).

Contrary to concerns regarding comparative validity across stability strata, our treatment algorithm mirrors real-world decision-making where intraoperative stability assessment dictates fixation strategy.

This study redefines therapeutic priorities in pediatric radial neck fractures: stability assessment supersedes traditional internal fixation recommendations. By showing that cast immobilization can achieve excellent outcomes for stable reductions in our series, we provide evidence for reducing unnecessary hardware implantation—aligning with ALARA (As Low As Reasonably Achievable) principles in pediatric radiation/anesthesia exposure (14). Preoperative CT scans adhered to ALARA principles, with radiation doses optimized to 0.1 mSv (15).

In summary, this study adds to the armamentarium of minimally invasive techniques for pediatric radial neck fractures. The stability-guided algorithm presented here—utilizing a simple percutaneous reduction followed by selective cast immobilization—offers a compelling alternative to more resource-intensive methods such as universal elastic nailing. This approach may be particularly attractive in settings with access to fluoroscopy but limited availability of advanced intraoperative imaging or specific implant systems. By leveraging dynamic intraoperative stability assessment, it provides a rational basis for avoiding hardware implantation and its associated burdens in a well-defined subset of patients, without compromising early radiographic and functional outcomes.

It is also worth noting that alternative minimally invasive strategies are available, such as retaining the percutaneous K-wire for 3–4 weeks postoperatively. This approach may represent a balanced compromise—offering enhanced stability without requiring a second procedure for implant removal under anesthesia—although it entails a theoretical risk of pin tract infection. A future randomized controlled trial comparing our stability-guided cast immobilization protocol with K-wire retention or universal elastic nailing would be highly valuable in definitively establishing the optimal treatment strategy.

## Limitation

This study has several limitations that should be considered when interpreting the results. First, its single-center, retrospective design with a modest sample size introduces the potential for selection bias and confines the generalizability of our findings. The treatment allocation was based on intraoperative stability, a clinically necessary but non-randomized process that precludes direct causal comparisons between techniques. The absence of a control group treated with alternative fixation methods further limits the comparative strength of our conclusions. Second, while functional outcomes were excellent at 6 months, the limited follow-up duration precludes assessment of long-term sequelae such as premature physeal closure or avascular necrosis, which typically manifest years post-injury. Finally, the economic implications, though suggestive of cost savings with PKWLR-CI, were not formally analyzed and warrant future investigation. Furthermore, the treatment allocation based on intraoperative stability introduces a fundamental element of selection bias. The PKWLR-EIMN group, by protocol, likely contained a higher proportion of inherently unstable fractures. Therefore, our findings should not be interpreted as a direct comparison of two equivalent cohorts, but rather as a validation of a stability-guided treatment algorithm. This algorithm successfully identified a cohort of fractures with sufficient inherent stability (the PKWLR-CI group) that could be effectively managed without internal fixation, thereby avoiding the burdens associated with implant removal.

## Conclusion

In conclusion, this study demonstrates that intraoperative stability is a reliable criterion for guiding treatment intensity in pediatric radial neck fractures. For fractures judged stable following percutaneous reduction, cast immobilization alone is a biomechanically sound and clinically effective strategy that spares patients and the healthcare system the morbidity and costs associated with unnecessary hardware implantation. These findings provide strong clinical support for a paradigm shift toward selective, stability-based treatment protocols in pediatric trauma care.

## Data Availability

The original contributions presented in the study are included in the article/Supplementary Material, further inquiries can be directed to the corresponding author.
